# Aesthetic Value of the Relationship between the Shapes of the Face and Permanent Upper Central Incisor

**DOI:** 10.1155/2010/561957

**Published:** 2010-09-08

**Authors:** Felipe de Oliveira Farias, Jussara P. Ennes, José Roberto Zorzatto

**Affiliations:** ^1^School of Dentistry, Federal University of Mato Grosso do Sul, 79070-900 Campo Grande, Mato Grosso do Sul, Brazil; ^2^Department of Morphophysiology, Federal University of Mato Grosso do Sul, 79070-900 Campo Grande, Mato Grosso do Sul, Brazil; ^3^Department of Computing and Statistics, Federal University of Mato Grosso do Sul, 79070-900 Campo Grande, Mato Grosso do Sul, Brazil

## Abstract

The relationship between the shapes of face and teeth has been analyzed for esthetic purposes and exploited commercially. The methodology to assess this correlation, described in the literature, does not enable reliable application of the concepts. Digital photos of face and incisors of 100 youths were measured with the software Image Tool 3.0 and classified as to shape. The same photographs were also analyzed and classified by a visual criterion. Statistical analysis of the metrical classification was performed by Pearson's correlation coefficient. The Kappa test was used to determine the coefficient of agreement between the observers and the obtained data, and to assess the agreement between both classifications (metrical and visual). The classificatory analysis made by the observers indicated a marked level of disagreement, also identified between their classification and that obtained by metrical analysis. The results indicated no relationship between the shapes of the face and the central incisor.

## 1. Introduction

The position, shape, and color of permanent upper central incisors enhance the esthetics of the smile. In cases involving the reconstruction of these teeth, parameters are required to assist in elaborating a plan of treatment that corresponds to the expectations of both patients and dental professionals [1]. 

Because of this demand, many methods have been proposed to determine the shape of central incisors. In 1914, Williams suggested a correlation between the inverted shape of the face and the shape of the upper permanent central incisors, the so-called “law of harmony" [2]. The contours of central incisors were classified into three categories*: tri*angular, oval, and square. Later, Frush and Fisher (1956) suggested a “Dentogenic Theory” that described the existence of a relationship between the shapes of the face and teeth together with gender and personality traits [3]. They believed, for example, that female characteristics such as smoothness and delicacy should be reflected in the teeth, through an oval shape and rounded borders. Likewise, male boldness and vigor should be expressed by square forms. However, neither of these studies used standardized methods to classify central incisor shapes.

Even today, there are authors who recommend the selection of artificial teeth or the determination of the shape of prosthetic reconstructions from a facial analysis [1, 4, 5], not only taking as a reference the shape of the face contour [1, 6], but also considering gender [5, 7]. However, there is neither consensus on such an approach nor a standard protocol for such an analysis.

Different methodologies have been suggested and employed to examine the existence of this possible relationship; moreover, instruments have even been developed based on this idea, such as the Trubyte tooth indicator [8]. Face shape has already been related to the inverted upper central incisor shape overlapping the outer contours, although without finding correspondence between shapes [9, 10]. The inaccuracy of human analysis in correlating the shapes of incisors and gender has also been emphasized [11, 12]. 

Some investigations have associated measurements of the face and the *central incisor*, with the main measures being the width of the zygomatic arch, the interpupillary distance, the distance between the inner corners of the eyes, the interalar width, and the distance between labial commissures (labial rima width) [8, 13–17]. These measures have been suggested as parameters to determine the central incisor width [13–17].

The absence of universal parameters to investigate these shapes leads to methodological empiricism and results in divergence as to the prevalence and reliability of analyses. Considering the inconclusiveness of past studies, this work sought to investigate the relationship between the face contour and the shape of the central incisor through both metric studies and visual analysis.

## 2. Materials and Methods

Photographs were taken of 100 young adults (mean age ± 20, in years), 40 men and 60 women, with whole central incisors. Individuals presenting any characteristics which could alter the contour of the face were excluded. Such characteristics comprised parafunctional habits, a background of face fractures, and facial plastic or orthognathic surgery. The research project was approved by CONEP (license number 1195 on 30/06/2008), and written consent was obtained from the participants. A digital camera (Sony DSC-F707 with Carl Zeiss Vario-Sonnar 9.7 mm zoom lenses) was placed at distances of 1.10 m from the face and 20 cm from the central incisor, in the frontal plane, with subjects seated in front of a black background. Images were edited with the help of the Photoshop CS 3 program initially by converting them to black and white; they were later measured using the Image Tool 3.0 program.

### 2.1. Metric Analysis of Face and Central Incisor

**Figure 1:**
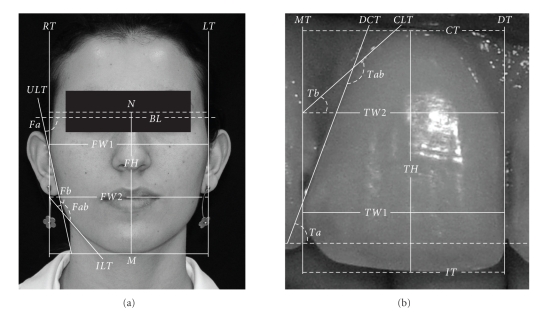
Measurements carried out to compare face and central incisors. Face: *FH*–face height, distance between nasion point (*N*) and menton base (*M*). *FW1*: maximum width of face, distance between right lateral tangent (*RT*) and left lateral tangent (*LT*). *FW2*: width of lower portion of face, at the height of the labial rima. *Fa*: angle determined by the tangent to the upper lateral face contour (*ULT*) and bi-pupillary line (*BL*). *Fb*: angle between the tangent to lateral lower contour (*ILT*) and the continuation of the line that determines *FW2*. *Fab*: angle formed by intersection of *ULT* and *ILT*. Incisor: *TH*: tooth height determined by the distance between the incisal tangent (*IT*) and the cervical tangent (*CT*). *TW1*: largest width of the incisor, distance between mesial tangent (*MT*) and distal tangent (*DT*). *TW2*: width of cervical third at the height of the limit of 1/3 of *TH*. *Ta*: angle determined by the tangent to the distal inciso-cervical contour (*CIT*) and by a virtual line parallel to the horizontal plane. *Tb*: angle formed by the tangent to the lateral cervical contour (*CLT*) and by the continuation of the line that determines *TW2*. *Tab*: angle formed by the intersection of *CIT* and *CLT*.

For measurement standardization, only the right incisor and the right side of each face were analyzed. In order to examine the relationship between face and central incisor, measurements were carried out (Figure 1) taking into account three criteria applicable to the images of face and central incisor contours. The face image was aligned keeping the bipupillar line (*BL*) parallel to the horizontal plane. The incisor image was aligned keeping the mesial surface of the tooth and the smile line perpendicular to the horizontal plane. These criteria allowed a geometric classification of faces and incisors.

Tangents were drawn at the farthest points of right and left face contours: the lateral longitudinal lines *RT* and *LT*, corresponding to the lateral mesial (*MT*) and distal (*DT*) tangents of the incisor. The greatest width of the face (distance between *RT* and *LT*) was denominated *FW1*, and the greatest width of the incisor (distance between *MT* and *DT*) was denominated *TW1*. The width of the face inner portion, at the height of the labial rima, was denominated *FW2*.

On the incisor, *TW2* corresponds to the width of the outer contour in the cervical third (at the limit of 1/3 of the tooth height). The incisor height (*TH*) was determined by the distance between the incisal tangent (*IT*) and the cervical tangent (*CT*).

The face height was determined by the distance between the nasion point (*N*) and the menton base (*M*). As the determination of the upper limit (hair line) is inaccurate and subject to large variations, the face upper third was not analyzed.

With the aim of comparing the general inclinations of face and incisor, three angles were determined. Firstly, angle *Fa* was determined by the tangent to the upper lateral contour of the face (*ULT*) and the bi-pupillary line (*BL*). Similarly, angle *Ta* of the incisor was determined by the tangent to the distal contour (*DCT*) and by a virtual line parallel to the horizontal plane. *Fb* represents the angle between the tangent to the inferior lateral contour (*ILT*) and the continuity of the line which determines *FW2*. On the incisor, *Tb* is the angle correlated to *Fb*, formed by the tangent to the lateral cervical contour (*CLT*) and by the continuity of the line which determines *TW2*. Finally, angle *Fab* was determined by the intersection of *ULT* and *ILT*, and, similarly, *Tab* was determined by the intersection of *DCT* and *CLT*.

The criteria used to compare face and incisor were (1) the *FH*/*FW* (face) ratio and the *TH*/*TW* (incisor) ratio; (2) the *FW1*/*FW2* (face) ratio and *TW1*/*TW2* (incisor) ratio; (3) angles *Fa* and *Ta*, *Fb,* and *Tb* and *Fab* and *Tab* of face and incisor, respectively.

### 2.2. Examiners' Classification of Face and Central Incisor Shapes

The digital archive of images was analyzed by three trained professionals (university professors of dentistry specialties, integrated clinics, and dental anatomy). Each examiner was given the images of faces and incisors (isolated) and was oriented to briefly observe each image and indicate the corresponding geometric figure. This classification was transcribed to a file card with a numerical identification of each image and a field for the geometric figure (square, triangular and oval), to be completed for face and tooth independently.

### 2.3. Statistical Analysis

Pearson's correlation coefficient was calculated for the face and the central incisor. To identify the degree (index) of agreement among the examiners, the Kappa test was applied [3, 18]. Finally, the same test was used to compare the classification obtained by the examiners and that obtained from the measurements. The confidence level was set at 95%.

## 3. Results

### 3.1. Metrical Analysis of Shape and Relationship between Face and Central Incisor

The correlation between the linear measures of *FH*/*FW* ratio (face) and *TH*/*TW* ratio (incisor) was 0.2637 (*P* = .008) which, although significant, is low. The remaining correlations presented no statistically significant values. 

The overall mean values of the angles were: *F* *a* = 79.76 ± 3,16, *T* *a* = 81.22 ± 4.42, *F* *b* = 48.76 ± 6.12, *T* *b* = 57.00 ± 6.60,  *F* *a* *b* = 149.24 ± 5.68, *T* *a* *b* = 156.33 ± 5.51.*Ta*, *Fb,* and *Fab* presented significant differences between the means of males and females (*P* < .05). The angles *Fa* (face) and Ta (incisor) were used to develop a criterion of classification into geometric shapes (triangular, oval, and square). 

The straight line of linear regression was calculated from the measurements of angles *Fa* and *Ta*, and hence the highest and lowest values for the measurements were determined, which were in turn equally divided into three groups (Table 1). These angles were related to the geometric figures so that the lowest values classified the sample as triangular, the intermediate values as oval and the highest as square.

The face shape identified in the sample as triangular accounted for 30% of subjects, with the oval shape accounting for 40% and the square for 30%. There was no statistically significant difference between the *Fa* averages of males and females (*P* = .28, Kruskal-Wallis).

A statistically significant difference (*P* < .05, Kruskal-Wallis) was found between the means of incisors in individuals of both genders. The oval shape predominated (male 42.5% and female 53.3%). Among males 40% presented a triangular and 26% a square shape whereas of the females 15% had a triangular and 31.7% a square shape.

### 3.2. Analysis of Face and Central Incisor Shapes by Observers

The free-marginal Kappa (K_[free]_) [3] obtained among the three examiners was 0.23 (*P* > .05) for the face analysis and 0.38 (*P* > .05) for the incisor analysis. Values higher than 0.70 can be considered good. Table 1 presents the examiners' classification of the geometric figures of face and incisors.

The Kappa statistic was used to analyze the agreement between face and central incisor shapes (Table 2). Only one examiner exhibited a satisfactory agreement (Kappa) between face and central incisor shapes, and the others were nonsignificant. The shapes of face and central incisor, obtained by the classification of angles *Fa* and *Ta*, did not obtain significant agreement (*P* > .05).

The assessment of agreement between the examiners and the metric classification (*Fa* and *Ta*) by the Kappa statistic (Table 2) indicated low Kappa values.

## 4. Discussion

The purpose of this paper was to identify face and central incisor shapes and to investigate the possible resemblance between the contours of face shapes and central incisors. This is a complex assessment because it analyzes two structures, face and central incisor, with very few elements in common. The principle of resemblance states: “two things are equal when they have everything in common; they are different when they have nothing to partake and similar when the common elements prevail over the differences” [19].

A reflection of this complexity is the disagreement between the examiners in the classification of face and incisor shapes (Tables 1 and 2). For the visual system, the more insufficient and ambiguous a visual stimulus is, the more its internal representation reflects the trends of perception of the observing mind. In this case, the observer's perception is more decisive than the characteristics of the stimulus [19].

Visual perception is characterized as being classificatory, meeting criteria to structure the stimuli and taking into account the proximity, similarity, closure, and continuity of elements (luminous stimuli) to determine shape. “Good shapes” are those that adapt to the laws of symmetry, continuity, proximity, simplicity, homogeneity, closure, and compactness. They tend to substitute for “bad shapes”, influencing esthetic preferences. Visual information is subject to the influence of the mind, which is not instinctively logical. For example, we allow our beliefs about the world to interfere with assessments; we tend to confirm more than doubt our hypotheses; we are unable to correctly use the principles of implication and to calculate probability. We consider an event more probable just because it is easier to imagine it so [20].

Neither the face nor the central incisors constitute geometric figures themselves. Thus, in ideal terms, we should use evaluation criteria that permit visual correlation with geometric figures. In the face, we find that the width of contour in the lower part (*FW2*) accompanies the inclination of the upper lateral tangent of the face (*Fa*); thus, if this inclination decreases, the face will also be narrower in the lower third. In the incisor, a point of contact marks the beginning of a more enhanced inclination of mesial and distal surfaces towards the cervix. Teeth can be observed with almost parallel proximal surfaces which incline markedly from the cervical third. This characteristic poses a complicating factor to the visual classification of the shape of the incisor.

Although the angles *Fa* and *Ta* more accurately represent the general inclinations of face and central incisor, comparison with the examiners' classification (Table 2) reveals marked disagreement that confirms the difficulty in visually associating the face and incisor with geometric figures [11, 12].

Although the correlation between the *FH*/*FW1* ratio (face) and the *TH*/*TW1* ratio (incisor) was significant, it was very low and insufficient (R^2^ = 0.06) to determine a relationship between the shape of the face and that of the central incisor.

Apart from these aspects we should consider some others related to the object of analysis. In this study, a young and healthy sample was evaluated, with no marked alterations in the shape of the face and incisors. There is a natural and considerable prevalence of facial asymmetry in the population [21] and great variability in face shapes. Diseases such as Cushing's Syndrome [22] and lipodystrophy [23] may alter the facial contour. They hamper the observation and determination of a common sense criterion as do several other factors including bodyweight, the use of jewelry, and age. With increasing age, a generalized subcutaneous dehydration occurs that contributes significantly to skin wrinkling and contraction, leading to a reduction of facial volume [24]. In some cases a decrease in the vertical dimension of the face also occurs, giving the face a slightly shorter aspect and making the cheek contour more prominent.

Clinically, several elements combine to determine tooth shape, such as color and contour of the reflective area, resulting from the interaction with light [25]. Furthermore, incisors have anatomic characteristics that vary in accordance with age. The teeth of elderly people present a higher frequency of rotations, inclinations in the mesiodistal direction, deviations from the midline, abrasion and gingival retraction [26].

The esthetic result of the incisor form does not depend on an agreement between face and tooth shapes, as changes in the smile are more relevant than the shape of the incisor itself. For example, the lack of an interdental papilla on an oval tooth gives it a triangular aspect. One way to overcome this would be to disguise the lack of a papilla with a subtle increase in cervical volume. This could point to a squarer characteristic, indicating that even when the shape of the incisor contour is altered, a satisfactory esthetic result is achieved. The most important aspect in this case would be not the shape of the incisor itself, but overall harmony in the result [27].

Understanding of the limitations on vision and interference of the mechanisms of visual perception should prompt the dental professional to search for a larger number of criteria to classify the shape of the central incisor, in an attempt to decrease the risk of error caused by misinterpreting what is seen, by deduction, induction, and other forms of subjectivity typical of the mental processes that guide visual perception.

In addition, the dental professional should search for more elements which may harmonize the tooth shape with periodontal and peribuccal structures and with the shape of the face as a whole. This harmony should meet the needs of both patient and professional. 

The sum of all these elements makes the classification of face and central incisor shapes more susceptible to failures. This study showed neither a metric relationship between face and central incisor nor data to support Williams' “law of harmony” [2] or “Dentogenic Theory” [3].

## Figures and Tables

**Table 1:** Criteria for classification of face and central incisor shapes by angles *Fa* (face) and *Ta* (incisor) and distribution of face and central incisor shapes according to the examiners' classification.

	Triangular	Medium	Oval	Medium	Square	Medium
*Criteria*						
Face (*Fa*)	<78.01°	76.17 ± 1.86	≥78.02°, ≤81.53°	79.72 ± 1.06	81.54°>	83.42 ± 1.37
Incisor (*Ta*)	<78.80°	75.29 ± 2.76	≥78.81°, ≤83.64°	81.65 ± 1.44	83.64°>	86.28 ± 2.02

*Classification of face*						
Examiner 1	24%		16%		60%	
Examiner 2	26%		41%		33%	
Examiner 3	37%		30%		33%	

*Classification of central incisor*						
Examiner 1	35%		15%		50%	
Examiner 2	25%		24%		51%	
Examiner 3	19%		10%		71%	

**Table 2:** Global agreement and kappa index for face and central incisor shapes and angles *Fa* (face) and *Ta* (incisor), and for the examiners' classification and the classification generated by measurements.

	Examiner 1	Examiner 2	Examiner 3
*Face and Central incisor shapes*			
Agreements Obs (%)	49	78	29
Kappa	0.23	0.67	−0.06
Examiners' average (1–3)	52		

*Examiners' classification and the classification by measurements*			
*Face (Fa)*			
Agreements Obs (%)	36	48	48
Kappa	0.04	0.22	0.22

*Central incisor (Ta)*			
Agreements Obs (%)	42	54	48
Kappa	0.13	0.31	0.22
